# Supplementary material for: H2AX C-Terminal Dipeptide Truncation: A Master Switch of the DNA Damage Response
Source: bioRxiv. 2026 May 11:2026.05.07.723272. Preprint. [Version 1] doi: 10.64898/2026.05.07.723272 (PMC13192945; doi:10.64898/2026.05.07.723272)
Supplement: Supplement 1 [file media-1.pdf]

Supplementary Data: H2AX C-Terminal Dipeptide Truncation: A Master Switch of the DNA Damage Response.

Faith M. Joseph[1,2], Matthew V. Holt[2], John M. Jerome[3], Linda Zhang[1,4], Ashley G. Boice[5], Patricia D. Castro[6], Sofía I. Aramburu[7], Ruhee Dere[5,8], Susan M. Rosenberg[2,7], David R. Rowley[3], Nicolas L. Young[2,3,5,†]

[1] Translational Biology and Molecular Medicine Graduate Program, Baylor College of Medicine, Houston, TX
[2] Verna & Marrs McLean Department of Biochemistry & Molecular Pharmacology, Baylor College of Medicine, Houston, TX
[3] Department of Molecular and Cellular Biology, Baylor College of Medicine, Houston, TX
[4] Medical Scientist Training Program, Baylor College of Medicine, Houston, TX
[5] Center for Precision and Environmental Health, Baylor College of Medicine, Houston, TX
[6] Department of Pathology & Immunology, Baylor College of Medicine, Houston, TX
[7] Department of Molecular and Human Genetics, Baylor College of Medicine, Houston, TX
[8] Department of Medicine, Baylor College of Medicine, Houston, TX

[†]Correspondence: Nicolas.Young@bcm.edu

Supplementary Data

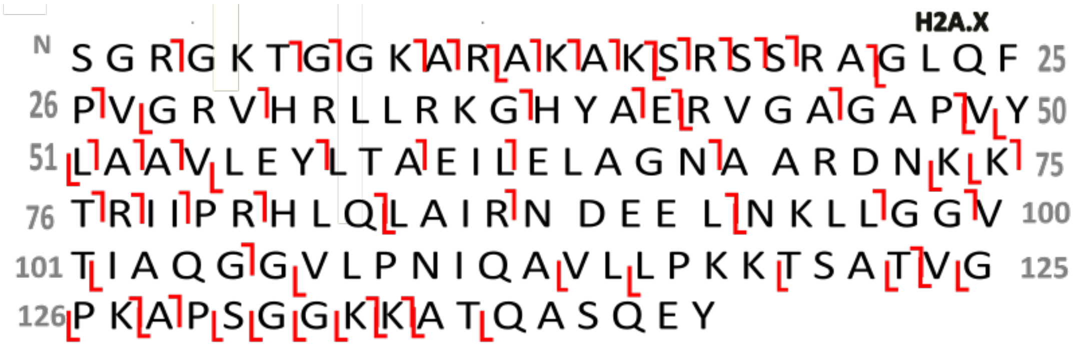

Supplementary Fig. 1 | Ion map showing the fragmentation pattern of full length H2AX in SUM159 cells.

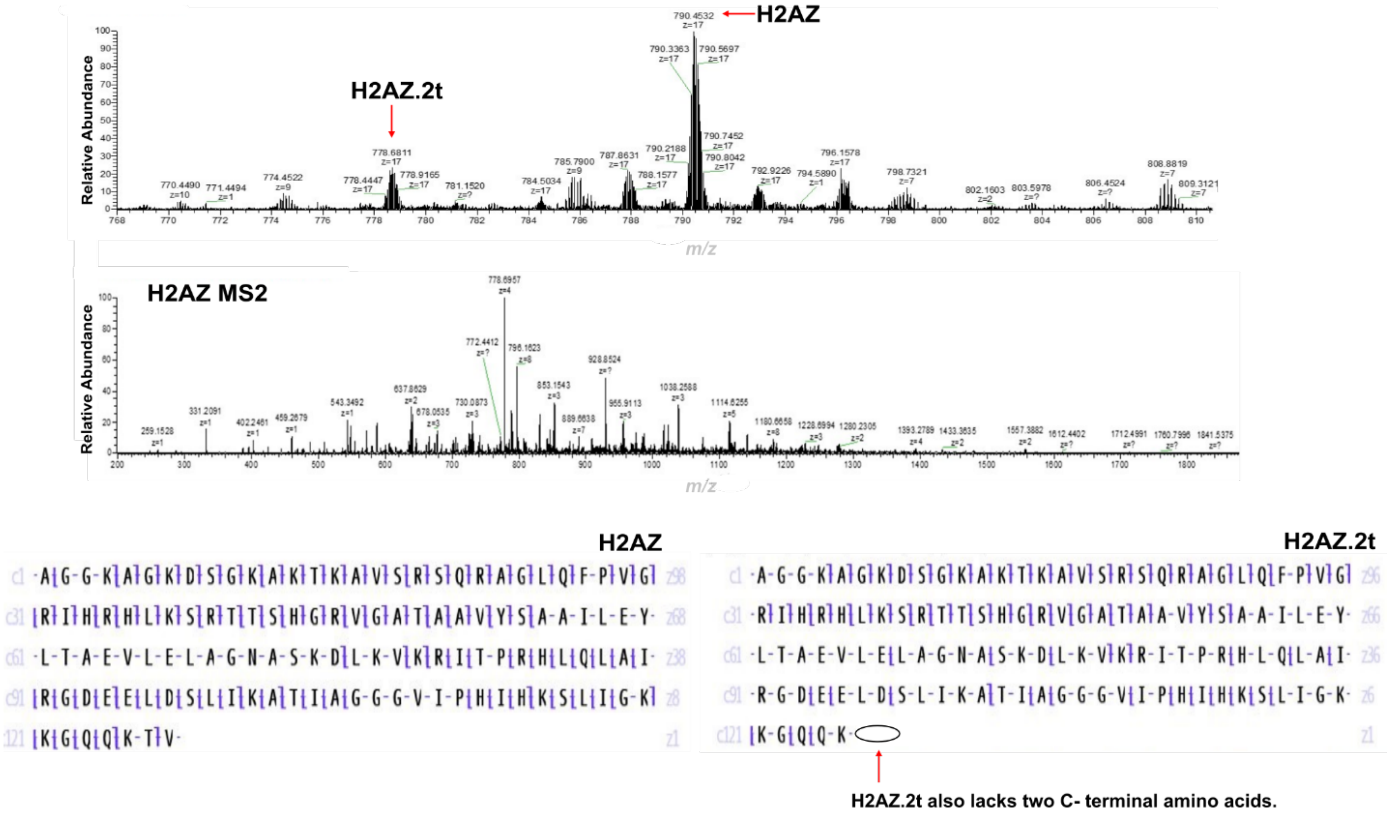

Supplementary Fig. 2 | Histone variant H2AZ.1 is also cleaved by two amino acids on its C-terminus.

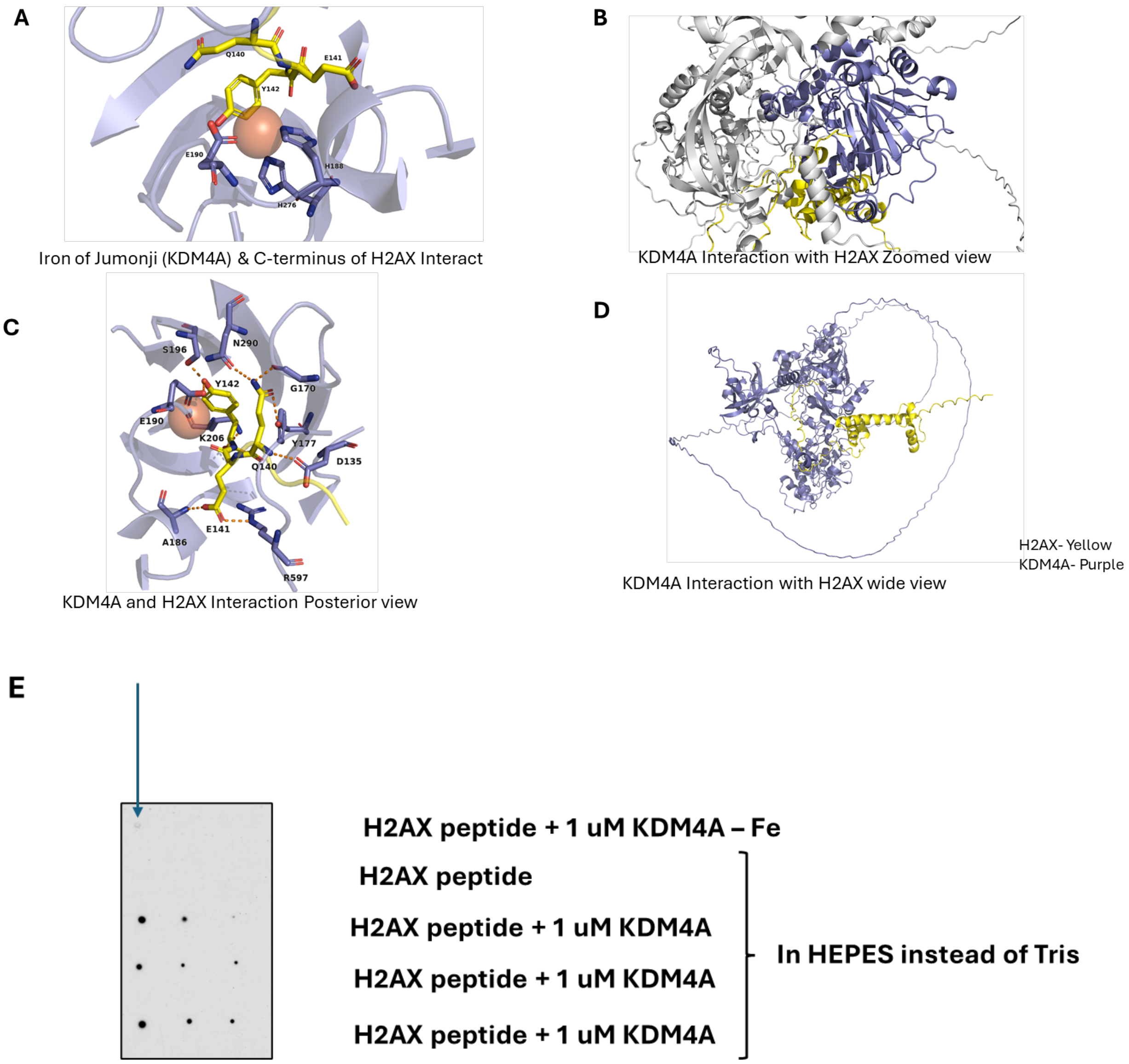

Supplementary Fig. 3 | Predicted Alpha Fold structure of full length H2AX, KDM4A interactions, and in vitro iron sensitivity. a, The C-terminus of H2AX is predicted to interact with the active site of the KDM4A Jumonji domains. The KDM4A contains an active site iron that is chelated by H188, H276, and E190. H2AX Y142 is predicted to directly interact with the active site iron, suggesting a mechanism of sequence specificity. This would bring the peptide bond between Q141 and E142, the site of proteolysis, in close unhindered proximity of the active site iron. **b-d)** The structured domains of full length H2AX and KDM4A are also predicted have complementary interactions in this configuration. **e**, Exclusion of iron during an in vitro reaction displays reduced activity between KDM4A and H2AX.2t (blue arrow). KDM4A and H2AX.2t activity is retained when the reaction is performed in HEPES instead of Tris buffer.

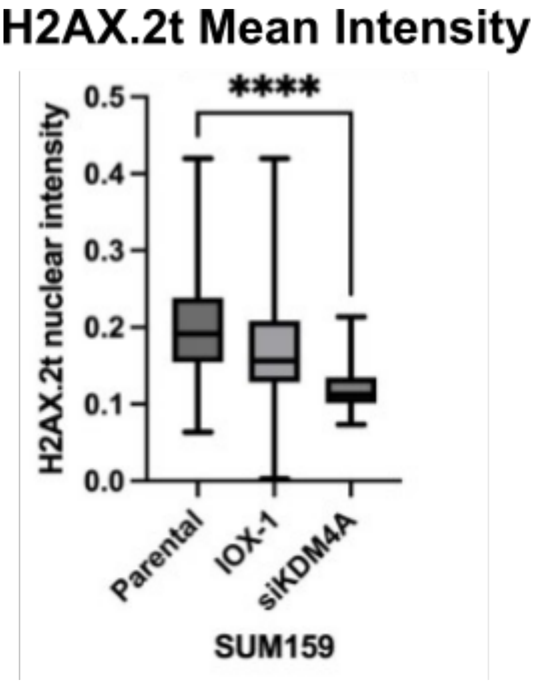

Supplementary Fig. 4 | Mean Intensity of H2AX.2t expression in SUM159 cells. Nuclear intensity of H2AX.2t is significantly reduced when KDM4A is knocked down in SUM159 cells.

Cohort

Cohorts: not selected

Results

P value: 2.9e-6

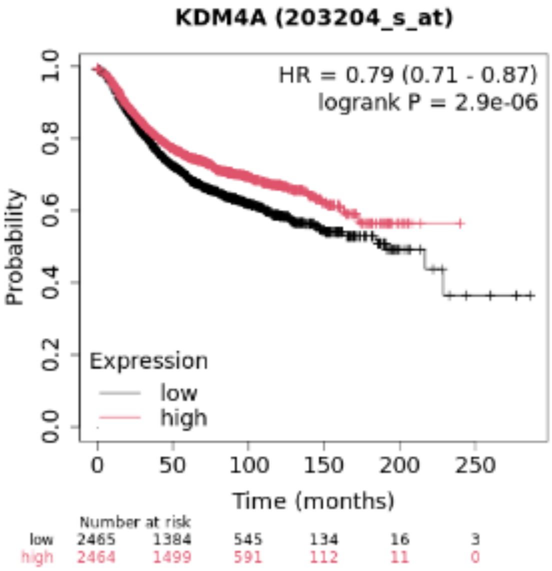

Download plot as a PDF

Upper quartile survival

Low expression cohort (months)	High expression cohort (months)
43	60

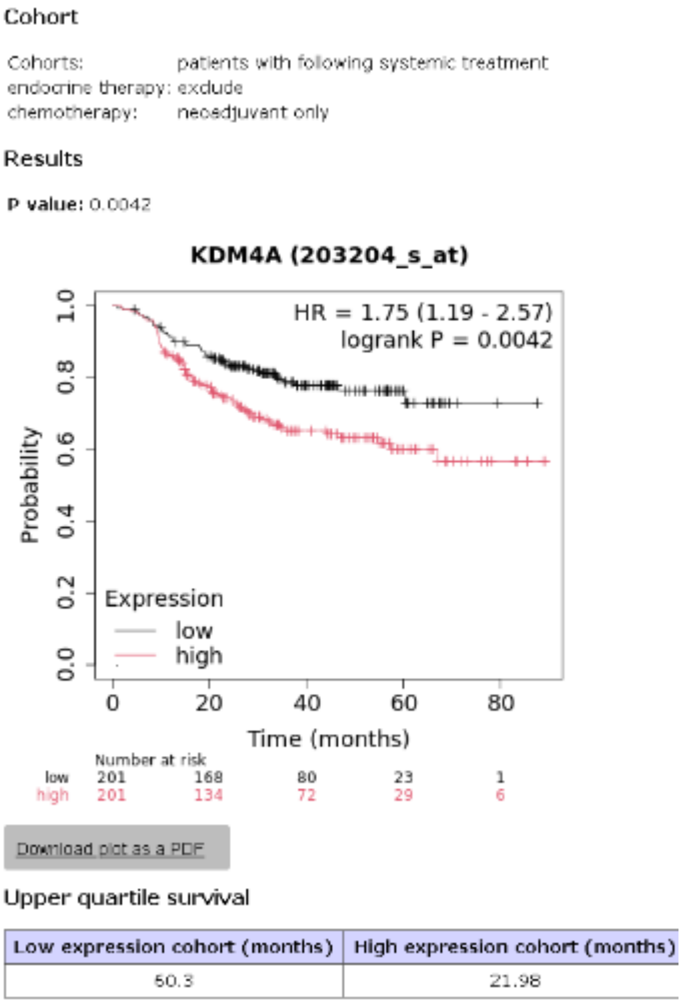

Supplementary Fig. 5 | KDM4A Cancer Prognosis. Above, km plot for breast cancer (all subtypes all treatments) for KDM4A is associated with better prognosis in breast cancer. Below, patients who only have neo-adjuvant chemotherapy have a worse prognosis when KDM4A is expressed.

Supplementary Table 1 | H2AX.2t expression is confirmed in the following human tissues

ID	TMA	ORGAN/TISSUE	DIAGNOSIS
60	5	LYMPH NODE	PAPILLARY CARCINOMA
756	5	OVARIAN	SEX CORD TUMOR WITH ANNULAR TUBULES

754	5	OVARIAN	GRANULOSA CELL TUMOR
753	5	TESTICLE	GRANULOSA CELL TUMOR
752	5	OVARIAN	GRANULOSA CELL TUMOR
738	5	RETROPERITONEAL	LIPOSARCOMA
720	5	CERVIX	NORMAL TISSUE
726	5	STOMACH	GASTROINTESTINAL STROMAL TUMOR (GIST)
716	5	LYMPH NODE	ADENOCARCINOMA
718	5	OVARY	DUCTAL CARCINOMA
526	3	LIVER	METASTATIC ADENOCARCINOMA
348	1	LYMPH NODE, INTRAPAROTID	MET MALIG MELANOMA
220	3	MENINGES	MENINGIOMA
306	2	LIVER	HEPATO CELLULAR CARCINOMA (HCC)
045	3	DURA MATER	MENINGIOMA
090	3	LYMPH NODE	POORLY DIFF ADENOCARCINOMA

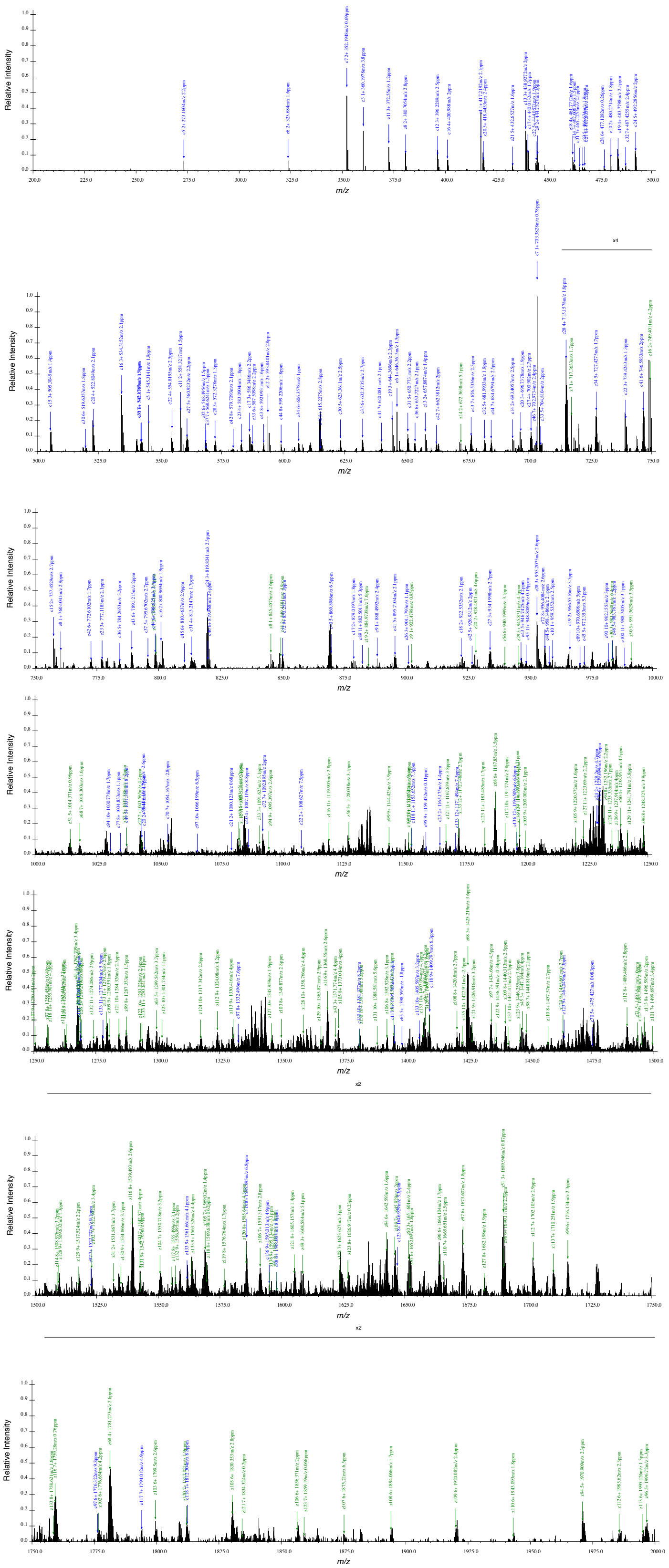

Supplementary Fig. 6. | Supplemental Figure 6. MS2 spectra of H2AX.2t in SUM159 cells

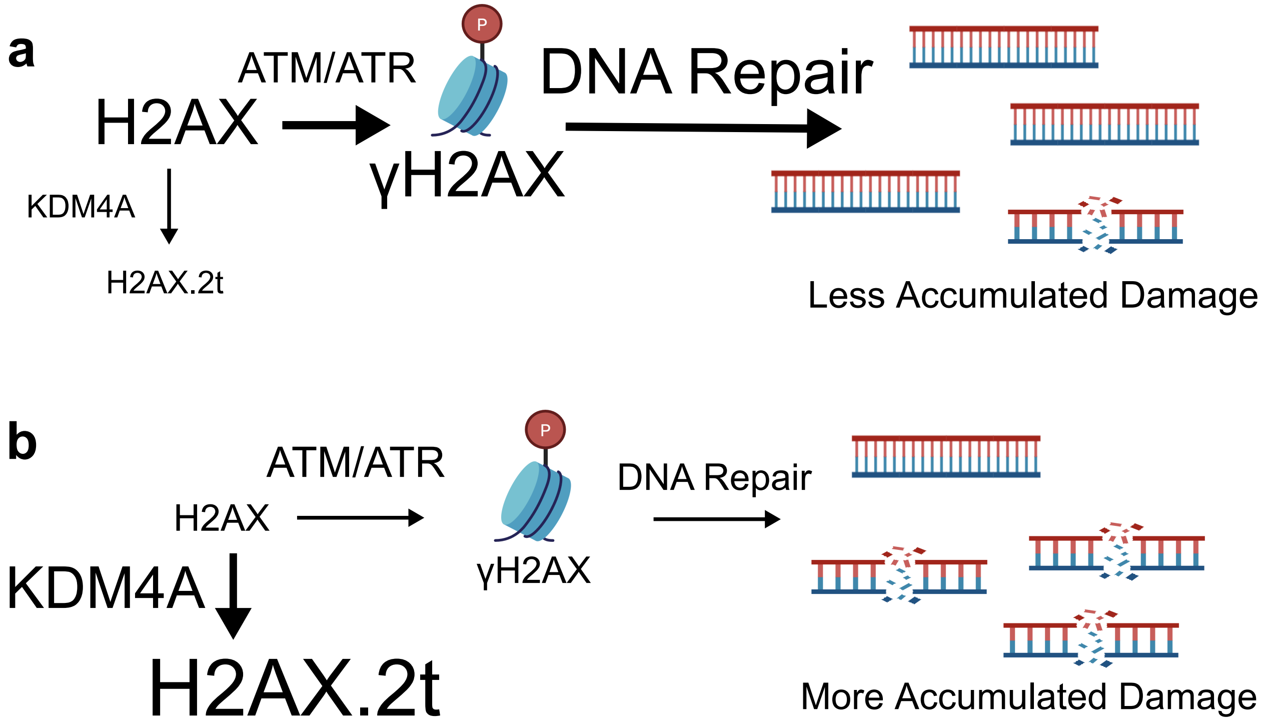

Supplementary Fig. 7. | How KDM4A activity affects H2AX function. a, Under normal conditions where KDM4A activity is low, there is sufficient H2AX available as substrate for ATM/ATR to signal for the DNA damage response. In this context, there is sufficient DNA repair to maintain genome stability and prevent DNA damage accumulation. **b**, When KDM4A activity is high, the pool of H2AX is depleted by conversion to H2AX.2t. Thus, there is less H2AX available as a substrate for ATM/ATR phosphorylation. This results in less DNA repair and more accumulated damage.
